# Increased Amygdala Activity Associated With Cognitive Reappraisal Strategy in Functional Neurologic Disorder

**DOI:** 10.3389/fpsyt.2021.613156

**Published:** 2021-03-26

**Authors:** Thomas Hassa, Stefan Spiteri, Roger Schmidt, Christian Merkel, Mircea Ariel Schoenfeld

**Affiliations:** ^1^Lurija Institute for Rehabilitation and Health Sciences, University of Konstanz, Konstanz, Germany; ^2^Neurological Rehabilitation Center Kliniken Schmieder, Allensbach, Germany; ^3^Department of Psychology, University of Konstanz, Konstanz, Germany; ^4^Department of Psychotherapeutic Neurology, Neurological Rehabilitation Center Kliniken Schmieder, Konstanz, Germany; ^5^Department of Neurology, Otto-von-Guericke-University Magdeburg, Magdeburg, Germany; ^6^Leibniz-Institute for Neurobiology, Magdeburg, Germany; ^7^Neurological Rehabilitation Center Kliniken Schmieder, Heidelberg, Germany

**Keywords:** functional neurologic disorder, conversion disorder, cognitive reappraisal strategy, fMRI, amygdala, emotion regulation

## Abstract

Cognitive reappraisal is an emotion regulation strategy to reduce the impact of affective stimuli. This regulation could be incomplete in patients with functional neurologic disorder (FND) resulting in an overflowing emotional stimulation perpetuating symptoms in FND patients. Here we employed functional MRI to study cognitive reappraisal in FND. A total of 24 FND patients and 24 healthy controls employed cognitive reappraisal while seeing emotional visual stimuli in the scanner. The Symptom Checklist-90-R (SCL-90-R) was used to evaluate concomitant psychopathologies of the patients. During cognitive reappraisal of negative IAPS images FND patients show an increased activation of the right amygdala compared to normal controls. We found no evidence of downregulation in the amygdala during reappraisal neither in the patients nor in the control group. The valence and arousal ratings of the IAPS images were similar across groups. However, a subgroup of patients showed a significant higher account of extreme low ratings for arousal for negative images. These low ratings correlated inversely with the item “anxiety” of the SCL-90-R. The increased activation of the amygdala during cognitive reappraisal suggests altered processing of emotional stimuli in this region in FND patients.

## Introduction

Patients with functional neurologic disorder (FND) present all varieties of neurological symptoms (1) that are not attributable to structural lesions. The neural correlates of this clinical condition are poorly understood. Early on, several etiological models of the disorder were proposed from a psychological point of view to fill this gap (2, 3). This is due not only to the missing somatic model of explanation but also to the fact that patients with FND exhibit distinctive psychological features (4) and striking common life events (5). Emotion processing plays a pivotal role in this context: epidemiologic data suggest that altered emotion processing is correlated with FND (6, 7) and a large number of imaging studies provide a wealth of evidence for altered emotion regulatory networks in FND (8).

The current neurobiological hypotheses concerning FND may be divergent (9–11); but the overlap of areas that are involved in emotion processing and simultaneously exhibit changes of activity in FND is remarkable (8, 12, 13). There is some evidence that the altered emotion processing may not just be a concomitant symptom but an aetiopathogenetic factor in FND. Diez and colleagues found that the functional connectivity of the amygdala to the anterior insula correlated with clinical improvement in FND (14) and also Espay et al. described changes of activation in the anterior cingulate cortex correlating with improvement of functional tremor (15). In a recent review the possible roles of emotional processing in generating and perpetuating FND symptoms were discussed (10); suboptimal emotional regulation, either over- or under-regulation, could, for example, affect (autonomic) arousal, emotional awareness and the interpretation of affective stimuli. Taken together, the reduced capacity to control the processing of emotions could be a keystone in the development of FND.

The most commonly investigated strategy to regulate emotion is cognitive reappraisal (16). Reappraisal uses the cognitive ability to evaluate a stimulus in a different context; it involves changing the way of thinking about a stimulus in order to change its affective impact (17), especially to reduce a negative impact. It has been shown that cognitive reappraisal is associated with healthier patterns of affect, social functioning and well-being (13). The reduced capacity to successfully apply cognitive reappraisal is a common feature across several major neuropsychiatric disorders (18). An incomplete downregulation of negative emotional impact could result in an overflowing emotional stimulation in FND. A raised level of emotion could favor perpetuating symptoms in FND patients. In a preceding study we found that an increased activation of the amygdala in FND by emotional stimulation was functionally connected to a symptom-specific neuronal network (19).

Concerning the underlying mechanisms it is commonly agreed that reappraisal recruits control regions to modulate emotional responses in the amygdala, even if it is still a matter of debate which of these control regions exerts a decisive influence. These regions include the dorsolateral and ventrolateral prefrontal cortex, the orbitofrontal cortex as well as the medial prefrontal cortex including the anterior cingulate cortex (13, 16, 20) and also the lateral temporal cortex and parietal regions (17). Likewise, it is generally accepted that reappraisal as a technique of downregulation should reduce the activation of the amygdala (20).

Based on these models we hypothesized that the neural correlates of reappraisal in FND should be different than in healthy controls. Assuming that the alteration of emotion processing was common to all FND patients independent of their clinical signs we included patients with a wide range of symptoms such as spastic or flaccid paresis, gait disorder, seizures, somatosensory disorder and dystonia. We applied a previously employed paradigm for cognitive reappraisal and performed functional magnetic resonance imaging (21). We expected patients with FND to have a higher activity than healthy controls in the amygdala during reappraisal indicative of an altered emotion processing in FND.

## Materials and Methods

### Participants

Twenty four patients (15 women, 9 men, with a mean age of 42,6 years ± 16,0; range 18,2–62,7 years; see Table 1) meeting the inclusion criteria of functional neurologic disorder (FND) according the DSM-V criteria were recruited consecutively. All patients underwent rehabilitation therapy in the Department of Psychotherapeutic Neurology for 3–10 weeks and were diagnosed by the same experienced clinician (RS). In all patients extensive neurological diagnostic procedures including MRI of brain and spinal cord, somatosensory evoked potentials, motor evoked potentials, peripheral nerve conduction examinations and EMG recordings were performed and did not reveal any pathological result. Patients with severe neurologic or psychiatric disorders including generalized seizures, post-traumatic stress or panic disorder, major depression or other major affective or psychotic disorders were excluded from the study.

**Table 1:** Demographic information and clinical symptoms.

**Patients**	**Age**	**Gender**	**Symptoms**	**Side**	**Duration**	**Controls**	**Age**	**Gender**
	**years**				**weeks**		**years**	
1	60	f	Monoparesis right arm	right	6	1	27	m
2	24	f	Paraparesis; Chronical pain syndrom	bilateral	158	2	50	m
3	61	m	Tics, Spasms, Somatosensory disorder	bilateral	61	3	39	f
4	59	m	Tics, Gait disorder	bilateral	612	4	30	f
5	61	f	“Choreatic” movement disorder; Halting speech, Dysarthria	bilateral	36	5	50	f
6	18	f	Gait disorder	bilateral	9	6	33	f
7	24	f	Paraparesis	bilateral	13	7	54	f
8	36	f	Monoparesis left leg	left	264	8	64	f
9	49	m	Tremor left arm	left	123	9	40	f
10	27	f	Paraparesis	bilateral	7	10	44	f
11	50	f	Hemiparesis, esp. Arm	right	4	11	46	f
12	29	f	Paraparesis	bilateral	23	12	23	f
13	54	f	Somatosensory disorder, Fluctuating monoparesis right arm	right	278	13	30	m
14	50	f	Monoparesis left leg	left	161	14	29	f
15	61	m	Hearing disorder	bilateral	83	15	39	f
16	20	m	Seizures, Fluctuating paresis	bilateral	9	16	48	m
17	31	m	Paresis left leg, Fluctuating paresis right leg	bilateral	210	17	54	m
18	52	m	Hemiparesis	right	6	18	46	f
19	49	f	Monoparesis left leg	left	3	19	40	m
20	62	f	Dystonia right arm	right	42	20	57	m
21	32	m	Paraparesis	bilateral	67	21	43	f
22	25	f	Monoparesis right leg	right	9	22	64	m
23	22	f	Chronical pain syndrom legs; Gait disorder	bilateral	615	23	64	m
24	56	m	Monoparesis left leg, Somatosensory disorder both arms	bilateral	63	24	61	m

*(f, female; m, male)*.

Age- and sex-matched control subjects were randomly recruited. None of the 24 (14 women, 10 men, with a mean age of 45,3 years ± 12,2; range 23,6–64,9 years) healthy controls had a history of neurological or psychiatric disease or any neurological deficits. The Ethical Committee of the University of Konstanz approved the study and all participants gave written informed consent.

### Self-Report Measure: Symptom Checklist-90-R (SCL-90-R)

All participants completed the German version of the SCL-90-R (22) [SCL90R-GSI (23)] as standardized questionnaire to identify general psychopathology and comorbidities. The SCL-90-R includes subscales to the items somatization, obsessive-compulsive, interpersonal sensitivity, depression, anxiety, hostility, phobic anxiety, paranoid ideation, psychoticism.

### IAPS Image Material

**Figure 1:**
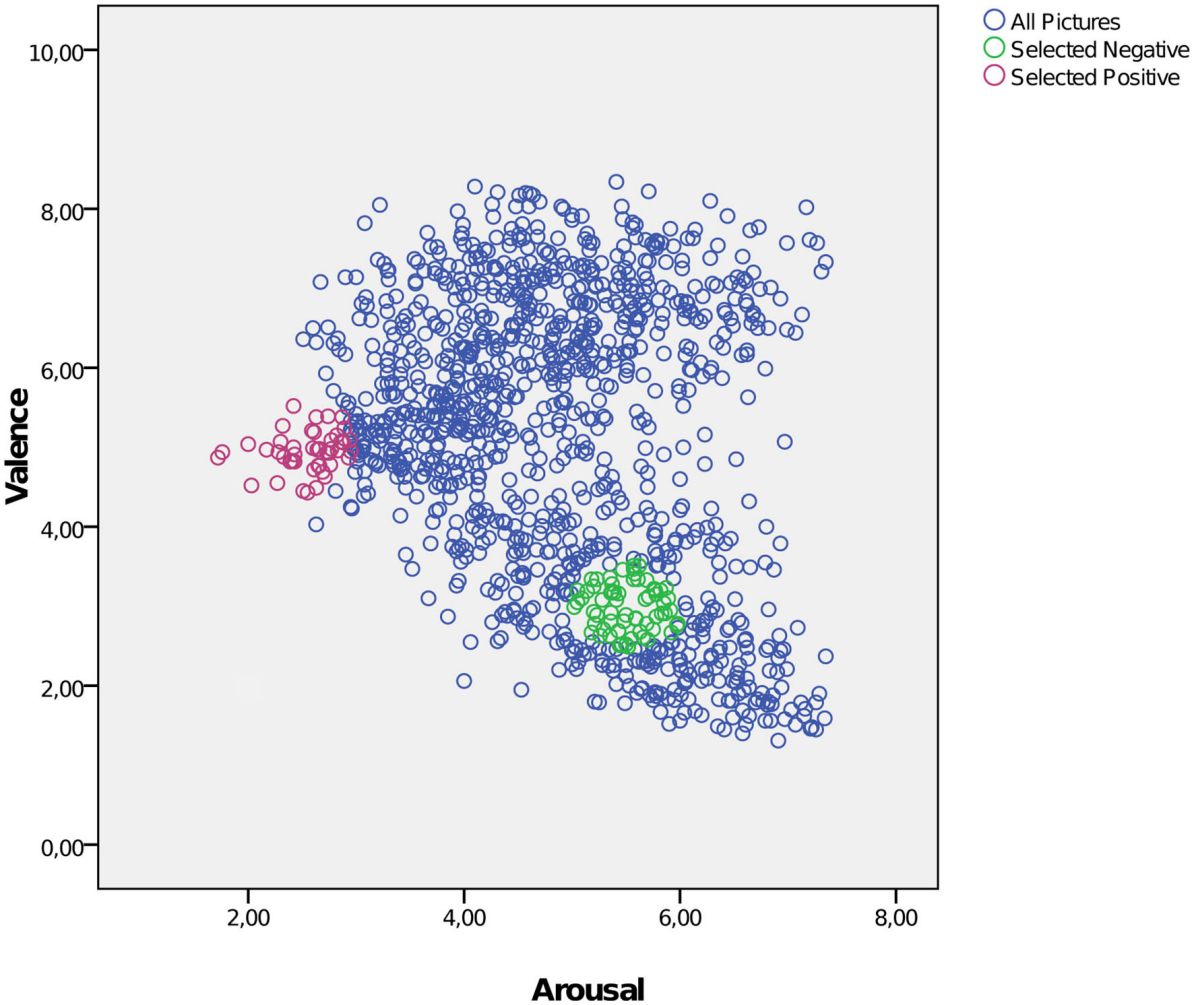
Selection of negative and neutral IAPS images intended to achieve a high uniformity with regard to the factors “valence” and “arousal.” Blue circles: all IAPS images; green circles: selected negative images; red circles: selected neutral images. X-axis: arousal; y-axis: valence (1 = very negative, 9 = very positive).

To induce emotional reaction for possible regulation we presented a set of images of the International Affective Picture System (IAPS, RRID:SCR_016869) (24) during the fMRI measurement. We selected images with a neutral or a negative valence expecting a clear effect of altered emotion regulation for the negative valence. Furthermore, the selection of images intended to generate high uniformity within the categories (negative/neutral) with regard to the factors “valence” and “arousal” in order to reach a high discriminatory power between the categories (see Figure 1). For negative images the specific value for valence was set at 3 (1=very negative, 9= very positive) corresponding to a higher degree of arousal of 5.5. For neutral images the specific value for valence was set at 5 and for arousal of 2.5. Within this framework we chose pictures located in close proximity to the specific values of valence and arousal from different thematic fields (violence, mutilation, war, animals, etc for negative images) to guarantee for a high heterogeneity of topics. At first glance the value selection for arousal of negative and neutral images seemed to be very conservative but it provided a high homogeneity for the different categories. According these criteria we selected 57 negative and 41 neutral images corresponding to the different account of events in the fMRI design for “negative” and “neutral.” The mean valence of the selected negative images was 2.996, range: 2.49–3.51; for neutral images 4.944, range: 4.43–5.52. The arousal of the selected negative images was 5.51, range: 5.02–5.98; for neutral images 2.55, range: 1.72–2.95.

The variance in brightness of the IAPS images is very high. To reduce visual effects associated with different degrees of brightness we changed the degrees of brightness of each image to an average value of 0.4 using the Gamma correction algorithm *pic*_*out* = *pic*_*in*^∧^(*log*(0.4)/*log*(*mean*(*pic*_*in*))).

### Emotion Regulation Task

To assess the neural correlates of emotion regulation subjects viewed neutral and negative IAPS images while being scanned in the fMRI. According to a paradigm used in a previous study with healthy subjects by Kanske (21), reappraisal was applied as cognitive strategy to reduce emerging emotions. The participants were instructed and familiarized with the task before the fMRI measurement outside the scanner. For reappraisal the subjects were instructed to directly find an explanation that could reduce the emotional impact of the negative image; for example, when seeing images presenting war or violence they suggested themselves that the scenes were not real but made-up movie scenes, that blood was not real but theater blood, etc. The reappraisal strategy had to be flexible considering the variety of images and themes. For this training version we used IAPS images different from the ones used in the fMRI session.

We presented negative images under 3 different conditions in a blocked fMRI-design. For condition (a) all participants were instructed to watch the negative image without any intervention to influence emerging emotions (NegW). During condition (b) participants regulated emerging emotions while regarding the negative image by using the previously taught cognitive reappraisal strategy (NegReapp). As distractive condition (c) (NegDistr) to control for the cognitive load of the reappraisal we projected a mathematical problem to be solved over the negative image. The arithmetical problems were formed with three operands including an addition and a subtraction (e.g., 8 + 9–6 = 13). The subjects were asked to decide for themselves without any feedback if the equation is correct or not. It was foreseeable that not all participants could solve the equations in the required period. To reduce the negative feeling of incompetence in failing they were instructed that it was not important for the study to succeed in arithmetic but to keep on calculating as long as the image was projected. The neutral images were used under two conditions to control for the emotional load: (d) watching the neutral images (NeutW) and (e) distraction by solving a mathematical problem (NeutDistr).

For stimulus presentation and MR scanner synchronization the software “Presentation” (Presentation, RRID:SCR_002521; http://www.neurobs.com, Albany, CA, USA) was used. Each image was presented for 7 s. After an induction period of 1,000 ms the instruction defining the different conditions was superimposed over the image for 1,000 ms. For the condition (a) and (d) (NegW and NeutW) the instruction “watch” was projected, for condition (b) NegReapp “reappraise”; for the distration condition (NegDistr and NeutDistr) the arithmetical equation. After the instruction the image remained on the screen in the execution phase for additional 5,000 ms. The events were interspersed with a jittered interstimulus interval of 3,000–8,000 ms (baseline) and they were arranged in a pseudorandomised order over 2 runs of 625 s respectively. Each of the three conditions with negative images occurred 19 times during the 2 runs resulting in 57 negative images whereby each single image was presented only once; the sequence of the images was randomized. The condition NeutW occurred 21 times, the condition NeutDistr 20 times, resulting in 41 neutral images.

Directly after the fMRI session the participants were asked to rate the 98 images of the study with regard to the valence and the arousal of each image using an analog scale from 1 to 10 (1: extreme low rating concerning emotion/arousal; 10: extreme high rating). As our set of stimuli included no positive images high values of valence reflected a high negative emotional impact.

### Image Acquisition

Magnetic resonance imaging was performed on a 3-T system (Skyra; Siemens, Erlangen) equipped with a 32-channel head coil for parallel signal reception. Functional T2^*^-weighted echoplanar imaging (EPI) was performed (36 axial slices of 3.0 mm thickness, no gap, FOV of 192 × 192 mm, 96 × 96 matrix, TR=2,500 ms, TE=30 ms, flip angle = 80°; parallel acquisition factor grappa=3; 250 volumes per session). A T1-weighted rapid gradient echo image (mprage: 1.0 mm iso-voxel; TR=2,700 ms, TE=7.21 ms) was acquired for co-registration and normalization. A high resolution FLAIR sequence (1.0 mm iso-voxel; TR=5,000 ms, TE=395 ms was acquired to exclude structural lesions.

### Statistical and Image Analysis

Behavioral data were analyzed using the Statistical Package of the Social Sciences (SPSS, RRID:SCR_002865) version 25 (SPSS Inc., Chicago, Illinois, USA). Non-normally distributed data were analyzed using non-parametric tests with the Mann-Whitney-*U* test for between-group comparisons. Correlations were calculated using Pearson's R for parametric and Spearman's Rho for non-parametric data.

Pre-processing and statistical analysis of the fMRI data were performed using the SPM12 software package (SPM, RRID:SCR_007037; Wellcome Department of Cognitive Neurology, University College London, UK) and MATLAB R2104a (MATLAB, RRID:SCR_001622; The Mathwork Inc.). The functional volumes were resliced, realigned to the first volume and spatially normalized to the EPI template in standard Montreal Neurological Institute (MNI) space. After resampling to a final voxel size of 2 × 2 × 2 mm, the spatially normalized images were smoothed with an isotropic 8 mm full-width at half-maximum Gaussian kernel and high pass-filtered (cut-off 128 s); movement parameters (six dimensions) from realignment were included as covariates into the model (25).

For statistical analysis, blood-oxygen level-dependent (BOLD) responses were modeled at the time of stimulus onset, i.e. the start of projection of the respective image and the duration of 7,000 ms. Due to the fixed temporal relationship of induction, instruction and execution phase it was not possible to disentangle the different phases. For each subject, the resultant regressors of main effects (NegW, NegReapp, NegDistr, NeutW, NeutDistr) were entered into a general linear model and convolved with the standard hemodynamic response function.

Group analyses were performed by submitting the individual-subject contrast estimates to a second-level; random effects over subjects were assessed in a mixed measures ANOVA design with the above mentioned conditions as a within subject factor and two groups (patients, controls) as a between subject factor. Correction for multiple comparisons on the second level was performed using a whole brain peak voxel threshold of *p* < 0.05, family wise error (FWE) corrected. In addition, small volume corrections (SVC) were performed in the amygdala, the medial prefrontal cortex including the anterior cingulate cortex (medPFC), the orbitofrontal cortex (OFC), the dorsolateral prefrontal cortex (dlPFC) and the ventrolateral prefrontal cortex (vlPFC) as regions of interest (ROI) related to FND (8, 12) and emotion regulation (13, 16, 20). For these regions we used bilateral masks from the automated anatomical labeling atlas (AAL, RRID:SCR_003550) (26). Activations were considered as significant if they survived *p* < 0.05 FWE corrected (SVC). The resultant activation maps were visualized using the MRIcron software package (MRIcron, RRID:SCR_002403; http://www.mricro.com).

### Analysis of the Time Courses

For all five main conditions the peristimulus time courses and the mean beta values were extracted from the amygdala on both sides of each single subject using the bilateral AAL- ROI of the amygdala (26) and marsBar (MarsBaR region of interest toolbox for SPM, RRID:SCR_009605) (27); the time courses and the mean beta values of all subjects of one group was used to calculate the mean average time course and the mean beta values of the group for each main condition.

## Results

### Behavioral Results

#### SCL90R-GSI

The mean value of the T-scores of the SCL90R-GSI for patients exceeded the threshold for the subscales somatization (T-score=65.67), anxiety (T-score =60.54) and the GSI (T-score =61.71); (T-score range of all items: 54.38–65.67); for controls all items were in the normal range <60 (range: 47.67–50.67).

Comparing the two groups the Mann-Whitney-*U* test revealed significant higher t-values for patients in 6 of 9 subscale items of SCL90R-GSI (somatization: z=-4.336, *p*=0.000, *r*=0.626; obsessive-compulsive: z=-2.76, *p*=0.006, *r*=0.398; interpersonal sensitivity: z=-2.52, *p*=0.012, *r*=0.364; depression: z=-2.705, *p*=0.007, *r*=0.390; anxiety: z=-3.656, *p*=0.000, *r*=0.528; psychoticism: z=-2.986, *p*=0.003 *r*=0.431) as well as in the global severity index GSI(z=-3.521, p=0.000, *r*=0.508), reflecting the well-known psychopathologies with FND (5, 28).

#### Rating of the IAPS Images

The IAPS image ratings concerning “valence” and “arousal” were not different between patients and controls (s. Table 2). Nevertheless, it was remarkable that the standard deviation and the range for negative images concerning valence and arousal were different, respectively the account of low ratings of negative images seemed considerably higher in patients. We tested if the account of extreme low ratings for negative images (1 or 1 & 2 on the analog scale 1–10) synonymous with a low impact of valence and arousal (s. Table 2) was different in both groups. The Mann-Whitney-*U* test showed a significant higher account of rating 1 (z= −2.15, *p*= 0.031, *r*=0.310) and rating 1&2 (z= −2.02, *p*=0.044, *r*=0.291) for arousal in patients; there was also a trend for a high account of rating 1 for valence (z= −1.85, *p* = 0.065, *r* = 0.267) in patients.

**Table 2:** Rating of IAPS images.

**Rating of IAPS images: mean values**						
			**Valence**		**Arousal**	
**Group**	**N**		**Neg images**	**Neutr images**	**Neg images**	**Neutr images**
**Patients**	24	mean (SD)	5.26 (±2.05)	1.71 (±0.63)	5.05 (±2.16)	1.55 (±0.84)
		range	1.23–7.98	1.00–2.95	1.00–8.70	1.00–4.83
**Controls**	24	mean (SD)	5.28 (±1.79)	1.64 (±0.49)	5.41 (±1.42)	1.46 (±0.45)
		range	1.89–7.88	1.15–3.05	1.93–7.81	1.02–2.83
**Rating of IAPS images: number of low ratings for negative images**						
			**Valence**		**Arousal**	
**group**	***N***		**number of rating 1**	**number of ratings 1&2**	**number of rating 1**	**number of ratings 1&2**
**Patients**	24	mean (SD)	7.54 (±12.19)	12.29 (±13.82)	10.04 (±13.37)	17.63 (±16.44)
		range	0–53	1–54	0–57	0–57
**Controls**	24	mean (SD)	4.46 (±7.44)	9.88 (±11.84)	3.50 (±5.60)	8.13 (±10.23)
		range	0–29	0–43	0–19	0–43

*neg: negative; neutr: neutral; SD: standard deviation; rating 1 or 1 & 2 on an analog scale 1–10 corresponding to a very low impact of emotional valence or arousal*.

In order to explore if the rating of the images was reflecting personal psychopathology in patients we further tested the correlation between the number of extreme ratings and the items of SCL90R-GSI. We found an inverse correlation for the high account of low rating value 1 and value 1&2 for arousal with the item anxiety (value 1: *r* = −0.428, *p* < 0.037; value 1&2: *r* = −0.460, *p* < 0.024).

### Imaging Results

**Figure 2:**
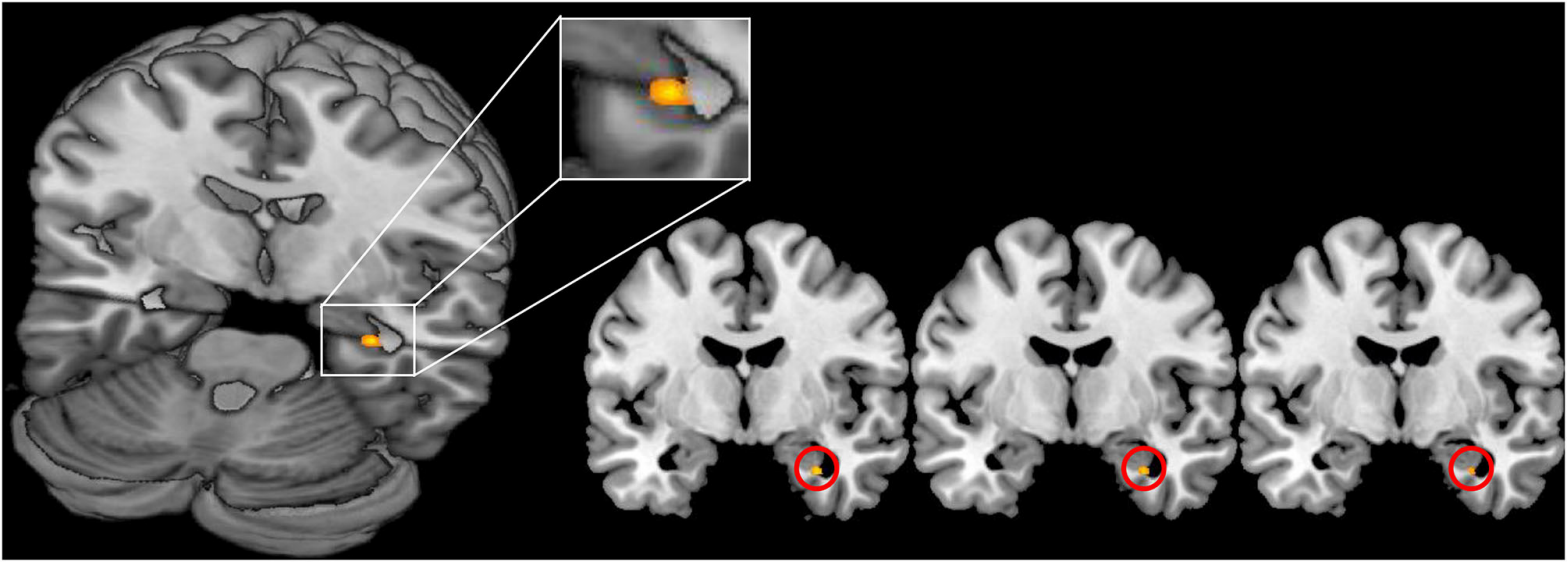
Increased activation of the right amygdala in FND patients vs. healthy controls during reappraisal (contrast NegReapp patients > NegReapp controls; MNI coordinates xyz= 30 −4 −28, Z=3.96, *p* = 0.039 cluster level FWE, small volume corrected). The activation is displayed on the MRIcron template.

For the main contrast NegReapp we observed extended activations in the bilateral dlPFC, vlPFC, OFC and the medPFC within both groups and also the differential contrast NegReapp>NegW showed activations in the left vlPFC and the medPFC within both groups. Comparing both groups with regard to the condition NegReapp the group × condition interaction showed significantly increased activation in patients compared to controls in the right amygdala (MNI coordinates xyz= 30 −4 −28, Z=3.96, T=4.03, kE 6 *p*=0.039 cluster level FWE, SVC; s. Figure 2). For the other main conditions NegW, NegDistr, NeutW and NeutrDistr and also the differential contrast NegReapp>NegW further group × condition interaction analyses did not reveal any increased activity, including small volume corrected analyses for the amygdala and the other predefined ROIs (medPFC, OFC, dlPFC and vlPFC).

To test the hypothesized downregulation in amygdala we reviewed the differential contrast NegW>NegReapp within both groups and analyzed both groups together (*n*=48). Against expectations we found neither activation in bilateral amygdala for NegW>NegReapp in patients nor in controls nor for all subjects together. This suggests that there is no modulation of activity in this area during reappraisal. On the contrary we observed a downregulation only during the distraction task: the contrast NegW>NegDistr revealed an activation in both amygdala for controls (right amygdala: MNI coordinates xyz= 26 −4 −16, Z=4.61, T=4.73, kE 121 *p*=0.001 cluster level FWE, SVC; left amygdala: MNI coordinates xyz= −24 0–20, Z=4.35, T=4.44, kE 129 *p*=0.001 cluster level FWE, SVC) as well as for patients (right amygdala: MNI coordinates xyz= 26 −4 −16, Z=3.45, T=3.50, kE 16 *p*=0.023 cluster level FWE, SVC; left amygdala: MNI coordinates xyz= −24 8–16, Z=3.96, T=4.04, kE 81 *p*=0.002 cluster peak level FWE, SVC). The group × condition (NegW>NegDistr) interaction showed no activation in both amygdala indicating no group differences in downregulation during the distraction task.

**Figure 3:**
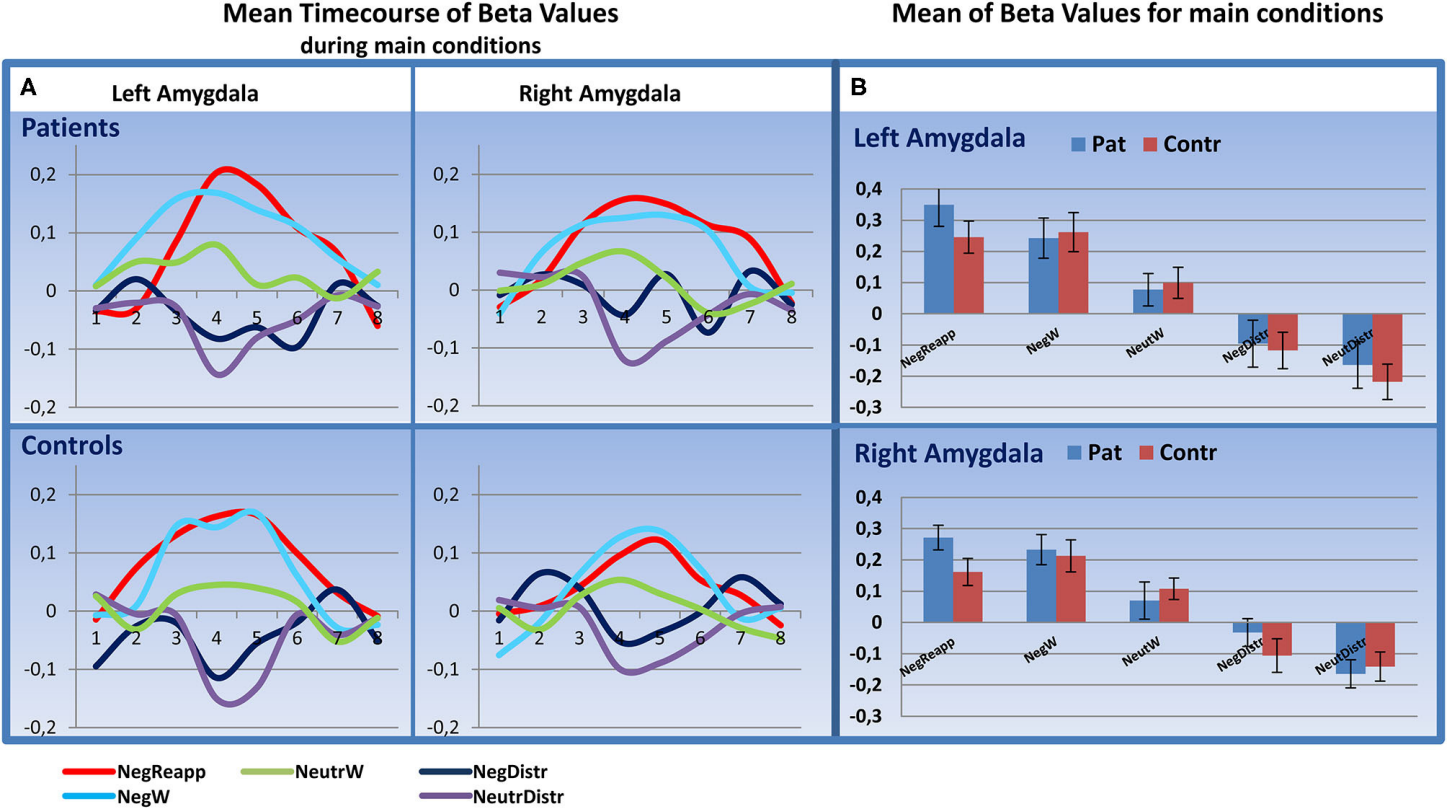
Time course analysis in the amygdala. **(A)** Mean Timecourse of Beta Values for 20 s (8 timebins à TR 2,500 ms after start of presenting the image) in the left and right amygdala: similar upregulation for NegReapp and NegW in both amygdala for patients and controls (*n*=24, each group). **(B)** Mean Beta Values with standard error of mean for all main conditions indicate as well an upregulation in both amygdala during NegReapp and a downregulation for the distraction conditions NegDistr and NeutrDistr. [main conditions: NegReapp: Reappraisal while watching negative images; NegW: watching negative images; NeutrW: watching neutral images; NegDistr: distraction condition (calculating) while watching negative images; NeutrDistr: distraction condition (calculating) while watching neutral images. TR: time repetition].

Analyzing the peristimulus time courses of activation and the mean beta values in the amygdala for all five main conditions we found a higher activity for NegReapp and NegW in bilateral amygdala for patients and controls (s. Figure 3). The distraction conditions NegDistr and NeutrDistr revealed a downregulation of activity in both amygdala.

## Discussion

The aim of this study was to investigate the neural correlates of emotional regulation in FND patients. According to current hypotheses cognitive control regions especially in the lateral prefrontal cortex attenuate activity in the amygdala during reappraisal (17, 20) resulting in a decreased emotional impact of the stimulus. It has been suggested that the function and connectivity of the amygdala in FND patients is altered. During emotional stimulation the activation of the amygdala is higher than in healthy controls (29); the resting state connectivity of the amygdala to various brain regions including emotion regulation and motor control circuits is increased in FND patients (30). Furthermore, a change of connectivity from the amygdala to the insula correlates with clinical improvement (14) and the increased activation of the amygdala is functionally connected to symptom-specific neuronal networks (19). These findings suggest an altered inhibition of emotionally-induced amygdala activity in FND. Here, the technique of cognitive reappraisal could be a valuable intervention to reduce the overshooting activity in emotion processing networks including the amygdala.

Due to the often reported augmented rate of traumatic life events in FND patients (6, 31) it could be hypothesized that the continuously increased activation in emotion networks could perpetuate symptoms in FND patients.

The results of the self-report measure (SCL-90-R) of our group of patients are well in line with the literature. The T-scores of anxiety and somatization exceeded the threshold in the group of patients and the group comparison revealed significantly higher *t*-values for patients in 6 of 9 subscale items of SCL90R-GSI (depression; anxiety; somatization; obsessive-compulsive; interpersonal sensitivity; psychoticism). This finding is related to the commonly reported psychopathologies with FND (28, 32, 33).

The analysis of the ratings of the IAPS images provided evidence for different behavioral subgroups of FND patients. The extent of extreme low ratings for arousal of negative images was significantly higher in the patient group in the absence of any difference for the means of the ratings. It can be assumed that one subgroup of patients rated extremely low either according to their lower extent of emotional awareness or to document an ostensible emotional stability. This was congruent with the clinical observation in these patients that the awareness of their own emotional involvement was low and that they tended to trivialize own emotional traumata. Furthermore, we found evidence that low-rating patients also showed correspondingly low values in the item anxiety of the SCL90R thereby underscoring this personal trait of psychopathology.

Already 2004 Reuber and colleagues (34) described subgroups concerning the personality inventory in a group of patients with psychogenic non-epileptic seizures (PNES). Brown et al. (35) found two clusters of patients with PNES, first a smaller cluster showing difficulties with most aspects of emotional regulation—including identifying, accepting, and describing feelings—and second a bigger group characterized by relatively high somatization and depression scores but comparatively normal levels of alexithymia. In a recent review the heterogeneity of patients with PNES is extensively discussed with respect to the levels of anxiety, alexithymia, emotional awareness and psychopathology (36). It can be assumed that the rating behavior in our study is reflecting these heterogeneities and that the patients who rated extreme low ratings for negative images show similarities with the first cluster in the study of Brown (35).

In line with actual models of emotion processing prefrontal control regions showed extended activations during emotional cognitive reappraisal, also controlled for the effect of watching negative images. But only the amygdala made a difference between the groups: we found an increased activation in the right amygdala of FND patients compared to controls during reappraisal. This finding underlines that the processing of negative emotional stimuli in FND patients during reappraisal is associated with a higher degree of activation in the amygdala than in normal subjects (37). This group effect was specific for the amygdala and the reappraisal condition; no other group × condition interaction showed a group effect in the amygdala or the other regions of interest (medPFC, OFC, dlPFC and vlPFC). For the activation in the right amygdala it was not possible to disentangle the effect of the cognitive act of reappraisal from just watching negative images. Nevertheless, these findings indicate that the downregulation of the amygdala during cognitive reappraisal was incomplete, or at least not fully effective in FND patients.

Contrary to expectations, there was no evidence of downregulation during cognitive reappraisal in both amygdala and in both groups: the time course analysis showed a clear upregulation (s. Figure 2) and the differential contrast of watching negative images vs. additional reappraisal revealed no different intensity of activation in bilateral amygdala independent of the emotional regulatory process. Most of the preceding imaging studies describe a downregulation of the amygdala during cognitive reappraisal (38–42).

The current result is different and in part contradictory to the previous findings. Insufficient statistical power cannot explain this finding: our group sample with 48 subjects in total is higher than in all quoted studies [highest sample in the study of Hayes et al. with *n*=25 (40)]. It cannot be excluded that the time of exposure to the negative images may have had some impact: in the study of Phan (42) the images were presented for 20 s, Goldin and colleagues (39) also stimulated for 15 s and observed a downregulation of activity in the amygdala only after 10 s. In order to generate a high uniformity and associated with this a high discriminatory power within the categories of images we chose not the maximum values for arousal and valence of negative images. It could be that the downregulation of the amygdala can merely be observed during high negative stimulation; Ochsner et al. (41) included only the maximum negative images in their analysis. Our stimuli had a comparable perceptual salience and a mid-to high but not the highest possible negative valence. However, the different result in our study points out to an important issue. The main neural mechanism by which cognitive reappraisal acts in FND patients is not necessarily the direct downregulation of overshooting activity in the amygdala. If such an effect is observable only after 10 s of presentation of very negative stimuli [see Goldin and colleagues (39)] it may rather be a secondary effect. With regard to the activity in the amygdala, in the current study the strongest reduction of activity was observed during distraction. However, there was no difference in the distraction-related modulation of amygdala activity between FND patients and healthy controls. If it is true that increased activity of the amygdala have a real impact on the development of FND then the idea of a cognitive control of emotions like in reappraisal do not seem to be an effective way to minimize this impact. It may be hypothesized that distraction in regard of emotional load could be a better strategy to deal with overflowing emotions.

In light of the current findings the concept of disinhibited activity in the amygdala in FND patients appears to be too simple. Taking the dynamic of connectivity changes between the amygdala and other cortical and subcortical regions into account might help solving the puzzle.

## Conclusion

In the current study FND patients exhibited an altered emotional processing which was reflected by particular arousal and emotional rating patterns of negative emotional pictures. Compared to healthy controls patients showed an increased activation of the right amygdala that did not decrease when cognitive reappraisal strategies were applied. Decreased hemodynamic activity in the amygdala was however observed during perceptual/cognitive distraction. This data suggests that cognitive reappraisal strategies primarily operate on higher neural processing levels different from the amygdala.

## Data Availability Statement

The raw data supporting the conclusions of this article will be made available by the authors, without undue reservation.

## Ethics Statement

The studies involving human participants were reviewed and approved by Ethical Committee of the University of Konstanz. The patients/participants provided their written informed consent to participate in this study.

## Author Contributions

TH, SS, RS, CM, and MAS conceptualized and designed the research. TH and SS performed the experiments. TH and MAS undertook the statistical analysis and wrote the first draft of the manuscript. TH, RS, and MAS interpreted the results and edited the final manuscript. All authors contributed to the article and approved the submitted version.

## Conflict of Interest

The authors declare that the research was conducted in the absence of any commercial or financial relationships that could be construed as a potential conflict of interest.
